# Comparison of the sensitivity of three cell cultures to ORFV

**DOI:** 10.1186/s12917-018-1760-1

**Published:** 2019-01-07

**Authors:** Guangxiang Wang, Yanhua Wang, Jiaqi Kong, Yanmin Li, Jinyan Wu, Yan Chen, Xiangtao Liu, Youjun Shang, Zhidong Zhang

**Affiliations:** 1 State Key Laboratory of Veterinary Etiological Biology, Lanzhou Veterinary Research Institute, Chinese Academy of Agriculture Science, Lanzhou, 730046 China; 2 National Foot and Mouth Disease Reference Laboratory, Lanzhou Veterinary Research Institute, Chinese Academy of Agriculture Science, Lanzhou, 730046 China; 3 Key Laboratory of Animal Virology of Ministry of Agriculture, Lanzhou Veterinary Research Institute, Chinese Academy of Agriculture Science, Lanzhou, 730046 China

**Keywords:** Orf virus (ORFV), Culture, Bovine Sertoli cells

## Abstract

**Background:**

Contagious ecthyma (CE) appears in the countries and regions containing goat and sheep farms, and it is considered a global epidemic. CE not only severely endangers the healthy development of the sheep and goat industries but also threatens human health. For viral infectious diseases, fast and effective isolation and culture of the pathogen is critical for CE diagnosis, and for disease prevention and control. Therefore, the sensitivity of bovine Sertoli cells to ORFV was estimate in this study.

**Results:**

The sensitivities of bovine Sertoli cells, primary neonatal bovine testicular cells, and Madin-Darby bovine kidney (MDBK) cell line to ORFV were compared. Our results showed that the isolated bovine Sertoli cells were sensitive to inoculated ORFV, and viral titers were approximately 1 log higher than those in primary neonatal bovine testicular cells and in MDBK cell lines.

**Conclusion:**

Appropriately sensitive cells for the highly efficient isolation and culture of the ORFV were obtained. Culture of ORFV using the Sertoli cells showed good consistency and stability and also avoided the risk of other pathogens presenting during viral culture using a primary cell line. In addition, using these passaged bovine Sertoli cells to proliferate ORFV may simplify the CE diagnosis process, thereby reducing detection time and cost. Hence, this test has important practical significance for the diagnosis of CE and the research on the pathogenic mechanism of ORFV.

## Background

Contagious ecthyma (CE), also termed contagious pustular dermatitis, is commonly known as “Orf”. It is a zoonotic disease caused by infection with a Parapoxvirus member, the Orf virus (ORFV), and it is an acute, infectious skin disease in humans, sheep, and goats that can be spread through contact. Infected goats and sheep usually have erythema, papules, boils, ulcers, and verrucous, thick calluses on the skin and mucosa of the lips, hooves, breasts, and vulvae [1–4]. CE has been classified as a reporting animal disease by the Office International des Epizooties and has been listed as a first-order animal disease in China.

CE was first discovered in Europe. It appears in nearly all countries and regions that contain goat and sheep farms [5, 6]. Existing epidemiological studies have shown an incidence of 60% and a mortality rate of 24.7% in adult sheep and goats, irrespective of anti-viral and antibiotic treatments [7], and the mortality rate in lambs was 93% [8]. Therefore, the incidence of CE causes significant economic losses for farmers and seriously endangers the healthy development of the sheep and goat industries. More seriously, this disease infects breeders through open wounds, and then virus multiplication causes telangiectasia and increase of capillary permeability resulting in exudation, finally forming herpes and ulceration on the dorsum of hands, the areas between the fingers, and on forearms [9–11]. For example, 8 breeders on a sheep farm in Yongan, Fujian Province, China contracted CE due to an ORFV infection in August 2005 [11]. In June 2013, a staff member at an animal disease prevention and control center in Jiangchuan County, Yuxi City, Yunna Province was accidentally bitten on the finger by an ORFV-infected sheep during sampling and photographing and became infected [12]. Thus, CE is a severe and dangerous zoonotic disease that not only endangers the healthy development of the sheep and goat industries but also threatens human health [13–16].

The rapid and effective isolation and culture of pathogens is critical to the diagnosis, prevention, and control of viral diseases. Cells that can permit viral replication are important tools for viral disease diagnosis and follow-up studies. ORFV can grow in the epithelial and kidney cells of cattle and sheep and in the testicular cells of calves and lambs, where they cause cytopathic effects (CPE) but display low viral titers. Recent studies have shown that the use of primary nasal turbinate epithelial cells from fetal sheep for ORFV isolation has multiple advantages, including convenient culture, high efficiency for viral isolation, and high titers of proliferating ORFV [17, 18]. However, primary cell collection from sheep embryos is a complicated procedure that requires numerous animals to provide sufficient tissue quantities for ORFV research [12]. A practical, simple, and reliable method for culture of ORFV is required. Thus, our work focuses on the research and development of passaged bovine Sertoli cells that are suitable for ORFV replication.

## Results

### In vitro growth behavior of bovine Sertoli cells at different temperatures

**Fig. 1:**
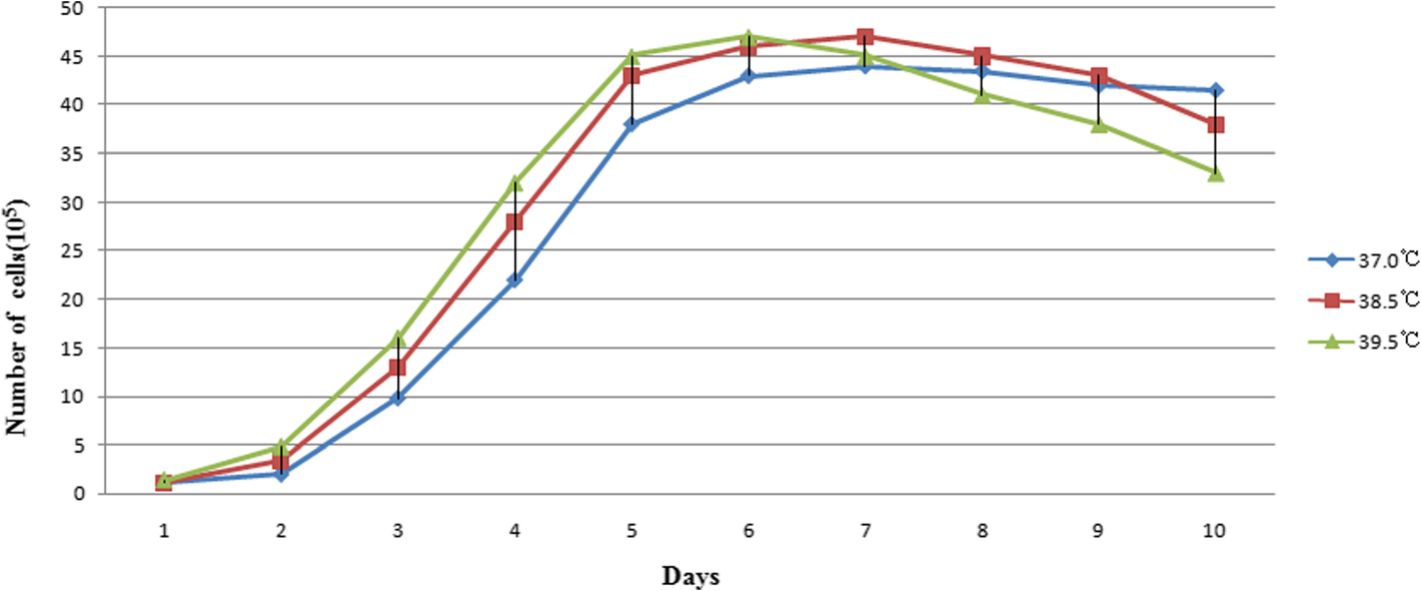
Growth curves of bovine Sertoli cells cultured in vitro at different temperature. The blue, red, and green curves represent the growth behavior of bovine Sertoli cells at 37 °C, 38.5 °C, and 39.5 °C, respectively

During culture at 37 °C and 38.5 °C, the interphase of bovine Sertoli cells was 2 d. Subsequently, the bovine Sertoli cells entered the exponential growth phase. When incubated at 39.5 °C, the interphase of bovine Sertoli cells was shorter than at 37 °C or 38.5 °C, and the bovine Sertoli cells entered the exponential growth phase earlier. Increased incubation temperatures also increased the replication rate of cells during the exponential growth phase. Irrespective of incubation temperature (37 °C, 38.5 °C, or 39.5 °C), the bovine Sertoli cells entered the plateau phase after 5 days of incubation. The plateau phase of the bovine Sertoli cell group grown at 39.5 °C only lasted approximately 1 d before the cells quickly degenerated and entered the decline phase. The bovine Sertoli cell plateau phase lasted approximately 4 d in the 38.5 °C group before entering the decline phase. In contrast, the bovine Sertoli cells in the 37 °C group remained in the plateau phase for the entire test period (Fig. 1).

### Measurement of bovine Sertoli cell morphology, HE staining, and indirect immunofluorescence staining

**Fig. 2:**
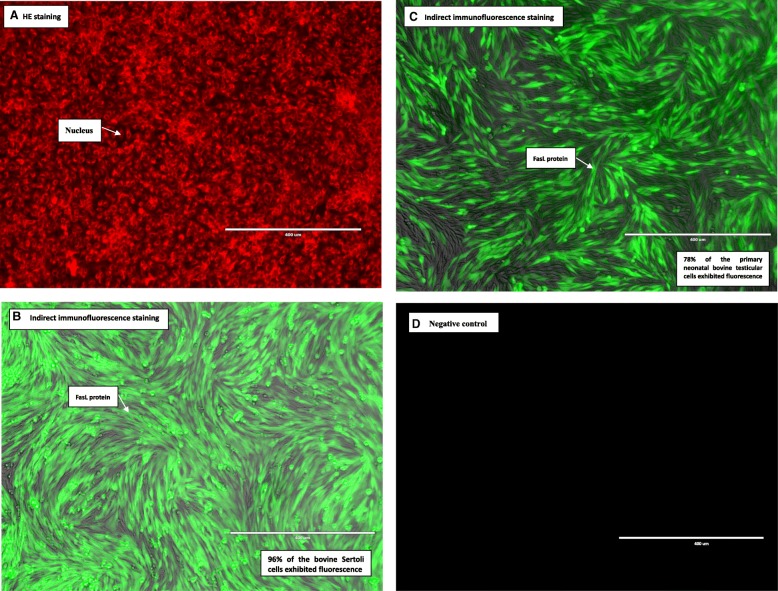
Identification results of the 3rd passage of the bovine Sertoli cells after isolation and purification by HE staining, and indirect immunofluorescence staining. HE staining assay (nucleus was indicated by 1 arrow) (**a**); FasL protein (indicated by 1 arrow) detection in the bovine Sertoli cells (**b**) and primary neonatal bovine testicular cells by immunohistochemical assay (**c**). Negative control (**d**). Bovine Sertoli cells and primary neonatal bovine testicular cells cultured for 96 h were visualized with bright-field and fluorescent microscopy, and the resulting images were merged. Ninety-five percent of the bovine Sertoli cells and 78% of the primary neonatal bovine testicular cells exhibited fluorescence, respectively

The bovine Sertoli cell bodies displayed a large cylindrical or irregular shape. Their cytoplasm spread widely and generally included 3 to 4 protuberances. The cell nuclei were oval in shape, localized at or near the center of the cell body, and contained 2 to 3 visible nucleoli. When multiple cells adhered to each other, their cellular protuberances interconnected with unclear margins, making the cells appear as a sheet-like monolayer (Fig. 2).

After HE staining, the cytomorphology of Sertoli cells was irregular, and the cytoplasm was fully spread. The cell nuclei were deeply stained, round or oval in shape, and localized at or near the center of cells. The cell nucleoli were prominent (Fig. 2a). Indirect immunofluorescence staining of the FasL protein in bovine Sertoli cell cultures showed that 95% of the isolated and purified cells were stained with green fluorescence (Fig. 2b and c).

After the isolation and purification of the culture, bovine Sertoli cells were finally obtained and submitted to the China Center for Type Culture collection (CCTCC) at Wuhan University (CCTCC accession number: C201438) for preservation.

### Comparison of the ORFV sensitivity of MDBK cells, primary neonatal bovine testicular cells, and bovine Sertoli cells

After co-incubating the 16th passage of the ORFV/HB/09 strain with the MDBK cells, primary neonatal bovine testicular cells, and bovine Sertoli cells for several hours, the CPE occurrence time, time to 75% CPE, and virus titers at 75% CPE (log TCID50/0.1 ml) were evaluated. As shown in Table 1, the CPE occurrence time, the time to 75% CPE, and virus titers at 75% CPE (log TCID50/0.1 ml) of bovine Sertoli cells were significantly different from the other 2 cell cultures.

**Table 1:** Sensitivities of the 3 cell cultures to ORFV

Cell cultures	CPE occurrence time (h)	Time to 75% CPE (h)	Virus titers at 75% CPE (log TCID50/0.1 ml)
MDBK cells	36.16 ± 0.29	96.00 ± 1.00	5.10 ± 0.10
Primary neonatal Bovine testicular cells	24.33 ± 0.29^a^	72.5 ± 0.5	5.50 ± 0.10
Bovine Sertoli cells	24.50 ± 0.53^*^	65.50 ± 0.50*	6.55 ± 0.58*

The bovine Sertoli cells from the 3rd passage were used in this experiment. Data are expressed as mean ± S.D. (*n* = 3, each group). Statistically significant differences between bovine Sertoli cells and MDBK cells and bovine Sertoli cells and primary neonatal Bovine testicular cells are indicated by *(*P* ≤ 0.05). The Mann-Whitney U test was used to compare the results of each group

^a^No differences were found between bovine Sertoli cells and primary neonatal bovine testicular cells

### Passaging of bovine Sertoli cells and its effect on ORFV replication

After the identification of the isolated and purified 3rd passage of bovine Sertoli cells, the bovine Sertoli cells were continuously passaged, while the cell growth time (to a confluent monolayer), time to 75% CPE after ORFV inoculation, harvested viral titer after passaging, and ORFV inoculation in each passage were recorded. The differences in ORFV replication between each passaged generation were recorded (Table 2).

**Table 2:** Bovine Sertoli cell growth properties and virus replication

Cell passage	Cell growth time (to a confluent monolayer, h)	Time to 75% CPE (h)	Virus titers at 75% CPE (log TCID50/0.1 ml)
3	72.16 ± 0.29	66.33 ± 0.29	6.47 ± 0.06
4	72.33 ± 0.29	65.50 ± 0.50	6.32 ± 0.03
5	72.50 ± 0.53	68.16 ± 0.29	6.35 ± 0.05
6	72. 33 ± 0.29	67.33 ± 29	6.42 ± 0.08
7	72.16 ± 0.29	69.50 ± 0.5	6.45 ± 0.05
8	72.16 ± 0.29	65.33 ± 0.29	6.35 ± 0.05
9	72.50 ± 0.53	65.50 ± 0.50	6.42 ± 0.03
10	78.33 ± 0.29	65.33 ± 0.29	6.37 ± 0.06
11	80.50 ± 0.50	70.00 ± 1.00	6.42 ± 0.08
12	85.33 ± 0.29	68.50 ± 0.53	6.40 ± 0.007
13	90.33 ± 0.29	66.33 ± 0.29	6.38 ± 0.104
14	92.50 ± 0.53	65.00 ± 1.00	6.35 ± 0.05
15	96.33 ± 0.29	69.33 ± 0.29	6.35 ± 0.05
16	105.16 ± 0.29	72.00 ± 1	6.00 ± 0.1*
17	105.67 ± 0.36	65.50 ± 0.50	5.9 ± 0.1*
18	110.33 ± 0.29	68.50 ± 0.53	5.80 ± 0.1*
19	120.33 ± 0.29	66.33 ± 0.29	5.52 ± 0.08*
20	120.50 ± 0.53	69.00 ± 1	5.13 ± 0.25*

Data are expressed as mean ± S.D. (*n* = 3, each passage). Statistically significant differences in different generations of passaged cells are indicated by *(*P* ≤ 0.001). The Mann-Whitney U test was used to compare the results of each passage

As shown in Table 2, the isolated and purified bovine Sertoli cells from the 3rd passage were continuously passaged until the 20th passage. As the passage number increased, bovine Sertoli cell replication was gradually reduced. The bovine Sertoli cells from the 19th passage took 5 d to reach a confluent monolayer, while the bovine Sertoli cells from the 15th passage required less than 4 d to reach a confluent monolayer. After the inoculation of the same passage of ORFV into different passages of cells, there were no significant differences in the time to 75% CPE (*P* > 0.5), and 75% CPE was observed from 65 to 70 h. However, the viral titers were significantly reduced in cells passaged more than 16 times (*P* < 0.001). In contrast, no significant difference in viral replication among different passages was found before the 15th passage.

## Discussion

To date, domestic studies of isolated testicular cells have been based on the method designed by Cameron et al [19] However, this method is complicated, contains multiple steps, requires a long operation time, and is not convenient for sterile operation. This study used the testes of neonatal calves as the test subjects and a combined enzymatic digestion (i.e., collagenase and trypsin) to prepare the single-cell suspension. Based on the rapid adherence of Sertoli cells and the slow adherence of spermatogonia, a differential adhesion strategy was used to isolate and purify the Sertoli cells. In this study, almost all of the Sertoli cells completely adhered to the flask wall after seeding for 6 h. Numerous non-adherent dot-like spermatogenic cells, epithelial cells, and mesenchymal cells appeared next to the adherent Sertoli cells. Therefore, the medium was changed 6 h after cell seeding to discard the non-adhered spermatogenic cells, epithelial cells, and mesenchymal cells during the few passages that showed rapid adhesion of Sertoli cells. This method simplified the isolation and purification steps of preparing a Sertoli cell culture and to a large extent avoided contamination with other cell types and with different stages of spermatogenic cells in the testes after sexual maturation.

Sertoli cells are one type of somatic cell in the seminiferous tubules. Compared with germ cells, Sertoli cells are less sensitive to temperature. In testis with cryptorchidism, due to the influence of temperature, the seminiferous tubules only contain spermatogonia, Sertoli cells, and a small number of spermatocytes [20]. Therefore, increased in vitro culture temperatures have been widely employed to isolate and purify Sertoli cells from adult animals. In this study, we compared the impact of 3 incubation temperatures (i.e., 37 °C, 38.5 °C, and 39.5 °C) on the adherence of Sertoli cells. Our results showed that the adherence of the Sertoli cell culture was better at 38.5 °C than at 37 °C, suggesting that during cell isolation in adult animals, increased temperature not only accelerates the apoptosis of spermatogenic cells [21] but also improves the cell adhesion of Sertoli cells. Van’t Hoff-Arrhenius temperature-coefficient equations predict that every 1 °C increase of body temperature accelerates basal metabolic rate and all chemical reactions by 20 to 30% [22]. In the present study, at an increased incubation temperature, although the replication rate of Sertoli cells in log phase was rapidly increased, their plateau phases were relatively short and unstable. At 39.5 °C, the plateau phase of the Sertoli cells lasted for only 1 d and was followed by rapid cell degeneration and entry to the decline phase, suggesting that although high temperature improves the adherence and replication of the Sertoli cells and accelerates the cell death of spermatogonial stem cells, it is not suitable for long-term in vitro cell culture. This study confirmed that the optimal temperature for the isolation and purification of the Sertoli cell culture was 38.5 °C. After the purification of the first 3 passages, an incubation temperature of 37 °C was optimal for continued Sertoli cell culture.

Currently, morphological observation and immunolabeling are typically used for the identification of Sertoli cells. Common and specific immune markers for immunolabeling Sertoli cells include vimentin, placental cadherin (P-cadherin), and FasL [23, 24].FasL is expressed by Sertoli cells in 2 forms, either membrane-bound or secreted. The distribution of Fas is rather broad, as many tissues and cell lines display weak expression of Fas. Among a variety of cell lines, only activated T cells and rodent Sertoli cells highly express FasL [25]. This study used HE staining combined with FasL indirect immunofluorescence labeling to identify Sertoli cells. Our results showed that FasL was highly expressed in 95% of cells, and the Sertoli cells obtained in this study were highly pure. Because in testicular tissue only Sertoli cells express FasL, positive FasL staining not only confirmed that the cells obtained from the cell culture were Sertoli cells but also indicated that the isolation method in this study did not damage natural FasL expression by the Sertoli cells.

ORFV can be cultured on nasal turbinate epithelial cells from fetal sheep, on a passaged bovine kidney cell line, and on calf and lamb primary testicular cells. In addition, neonatal or fetal bovine muscle cells, neonatal or fetal sheep hypodermal cells, fetal bovine pulmonary cells, fetal sheep spindle-shaped cells, and fetal sheep muscle cells can be used in culture for ORFV isolation [26–28]. Among those cell types, the MDBK cell line is the most commonly passaged cell line and is used for the initial viral isolation and subculture. Primary neonatal bovine testicular cells are commonly used in the culture and subculture of ORFV for isolation and attenuation and for the production of attenuated ORFV vaccines [28]. This study inoculated virulent ORFV (of the same passage number) into MDBK cells, primary neonatal bovine testicular cells, and isolated and purified bovine Sertoli cells to test and compare them in parallel. The isolated and purified bovine Sertoli cells were the most sensitive to ORFV among the 3 tested cell cultures and showed the highest viral replication rate (see Table 1). After the bovine Sertoli cells were isolated and purified, they were continuously subcultured until the 20th passage. As the passage number increased, the Sertoli cell replication rate gradually decreased, with a decrease in viral replication once cells were subcultured beyond the 16th passage. Nevertheless, the first 15 passages of cells grew well (required only 4 days to reach a confluent monolayer), and no significant difference in viral replication among different passages was found before the 15th passage. Compared with the primary neonatal bovine testicular cells that have been commonly used for ORFV culture, the first 15 passages of the isolated and purified bovine Sertoli cells showed more consistent parameters with respect to ORFV, such as the incidence of CPE, the time to ORFV harvest, and the viral titer. Therefore, the bovine Sertoli cells were ideal for ORFV culture.

## Conclusions

The use of the bovine Sertoli cells for the culture of ORFV ensures viral homogeneity and stability. Moreover, it may avoid the risk of contamination with other pathogens that may occur during viral culture from multiple preparations of primary cells. In addition, the use of the bovine Sertoli cells for ORFV culture may simplify the diagnosis process, shorten the detection time, reduce detection costs, and guarantee detection stability. This method thus has great practical significance for large-scale diagnosis.

## Methods

### Experimental animals and viral strains

Three one-day-old healthy male neonatal calves (35–45 kg) delivered by healthy cows (i.e., physically healthy with negative test results for foot-and-mouth disease, brucellosis, paratuberculosis, and bovine viral diarrhea) were purchased from a Baiyin dairy. The Madin-Darby bovine kidney (MDBK) cell line and ORFV Hubei virulent strain (ORFV/HB/09) were maintained and provided by our laboratory.

### Preparation of primary neonatal bovine testicular cells

All trials were conducted with the approval of the Ethical Committee of the Lanzhou Veterinary Research Institute of the Chinese Academy of Agricultural Sciences. Neonatal calves were fully anesthetized with isoflurane inhalation anesthetic administered by face mask (1 to 2% isoflurane) followed by exsanguination as an adjunctive method of euthanasia. These animals were placed in dorsal recumbency on the surgical table and prepared for placement of vascular introducer sheaths. Exsanguination was accomplished via an incision of the carotid arteries with a pointed, very sharp knife with a rigid blade at least 6 in. long and conducted as soon as the loss of consciousness was confirmed. Scrota were collected from euthanatized neonatal calves (scrotal root ligation was performed, and the scrotal surface was disinfected using 75% ethanol). Preparation of primary neonatal bovine testicular cells was performed as described by Zhou [29] with some modifications. The testicular parenchyma were collected and cut into uniform small tissue pieces by ophthalmic tweezers and scissors under sterile conditions. The small tissue pieces were then put into D-hanks solution and pipetted gently several times. After standing for 5–10 min, the supernatant was removed, and the convoluted seminiferous tubules were obtained. The convoluted seminiferous tubules were placed into 50-mL centrifuge tubes along with 10 volumes of lysis buffer containing 0.1% IV collagenase (GIBCO) and 0.25% trypsin (GIBCO), and the suspension was allowed to digest at 4 °C for 12 h or overnight. An equal volume of DMEM medium (HyClone) containing 10% fetal calf serum (GIBCO) was then added to stop the enzymatic digestions. After gentle pipetting several times using a Pasteur pipette, the tissue lysate was filtered with a 200-mesh copper wire screen. The digested solution was then collected and centrifuged at 1000 rpm for 10 min. The supernatant was discarded, and the pellet was washed twice with serum-free culture medium. The supernatant was again discarded, and high-glucose DMEM medium containing 10% fetal bovine serum was added to resuspend the cells. Trypan blue staining was used to determine cell viability. Then, the cells were seeded into a culture flask at a density of 1–2 × 10^5^ cells/mL and were incubated at 37 °C in a humidified incubator with 5% CO_2._

### Bovine Sertoli cell isolation, purification, and growth curve preparation

Bovine Sertoli cell isolation and purification were performed as reported [30, 31] with some modifications. The prepared primary testicular cell suspensions were seeded into culture flasks at a concentration of 1–2 × 10^6^ cells/mL and divided into 3 groups (5 flasks per group) that were then incubated separately at 37 °C in humidified incubators with 5% CO_2_ for 6 h. After incubation, the culture media containing non-adherent cells were discarded, and high-glucose DMEM culture medium containing 10% FBS was added to continue purifying the bovine Sertoli cells and to subculture the cells for 3 passages.

After the combined enzymatic digestions, the bovine Sertoli cells from the 3rd passage were seeded at a concentration of 1 × 10^5^ cells/mL into 24-well plates per group (100 μl per well), and the groups were incubated separately at 37 °C, 38.5 °C, and 39.5 °C in humidified incubators with 5% CO_2_. Three wells of cells from each group were collected every 24 h, starting from the initial seeding. The numbers of adherent bovine Sertoli cells in the 3 wells were counted using a hemocytometer and averaged to obtain the mean value. These steps were repeated for 10 consecutive days to determine the growth conditions of the bovine Sertoli cells at different temperatures. The optimal incubation temperature for purifying the bovine Sertoli cells was obtained based on the differential adherence.

### Identification of bovine Sertoli cells

Hematoxylin and eosin (HE) staining was performed on the bovine Sertoli cells from the third passage to observe their cell morphology. Fas ligand (FasL) protein in bovine Sertoli cells was detected by immunohistochemical assay. HE staining was performed by using a kit according to the manufacturer’s instructions (Beijing Solarbio Science and Technology Co., Ltd., Beijing, China).

For immunohistochemical assay, 3-day cell culture samples were fixed in a freshly prepared 50 g/L paraformaldehyde solution for 10 min and were then rinsed in phosphate-buffered saline (PBS) 3 times for 5 min each. Cellular antigens were blocked with normal goat serum at room temperature for 20 min. Rabbit anti-rat FasL polyclonal primary antibody (Abcam, 1: 100 dilution) was incubated with the cells at 37 °C for 3 h. After rinsing the cells with PBS 3 times for 5 min each, a fluorescein isothiocyanate (FITC)-labeled goat anti-rabbit IgG secondary antibody (Abcam, 1:50 dilution) was incubated with the cells at 37 °C for 30 min. The cells were then rinsed with PBS 3 times for 5 min each. The stained cells in 10 randomly selected fields were observed under a fluorescence microscope. The number of FasL-positive cells and the total number of cells in each field were calculated. The percentage of positive cells was calculated to determine the purity of the bovine Sertoli cells. PBS replaced FITC-labeled goat anti-rabbit IgG secondary antibody as the negative control.

### Comparison of ORFV sensitivities in MDBK cells, primary neonatal bovine testicular cells, and bovine Sertoli cells

The culture media on the confluent monolayers of MDBK cells, primary neonatal bovine testicular cells, and bovine Sertoli cells in culture dishes (3 flasks per cell cultures) were replaced by fresh media. Next, a 0.1 multiplicity of infection (bovine Sertoli cells were used for the titrations) of the ORFV/HB/09 strain virus (16th passage) was inoculated. After incubation for 30 min to allow complete adsorption of the virus, DMEM solution was added, the pH was adjusted to 7.0 to 7.2, and 100 units/mL of penicillin and 100 units/mL of streptomycin were added. The samples were incubated at 37 °C in humidified incubators with 5% CO_2_ for 40 to 72 h. The cell growth and cytopathic effects (CPE) were observed daily. When the CPE reached 75%, the viruses were harvested by 2 cycles of freezing at − 20 °C and thawing. The Reed-Muench method was used to measure the TCID_50_/0.1 ml value, representing viral replication in the 3 different cell cultures. One flask served as a control.

### Bovine Sertoli cell passage and ORFV replication assay

The isolated and purified bovine Sertoli cells continued to be passaged (each passage of cells underwent combined enzymatic digestion and was passaged at a 1:2 ratio). Each passage of cells was grown to a confluent monolayer (10 ml of culture medium in a 25-ml culture flask) before inoculation with a 0.1 multiplicity of infection of the 16th passage of ORFV/HB/09 strain virus (to obtain a total of 4 flasks of viral cell culture, in which 1 flask was used as control) to further test cell passaging (see previously described methods). The cell growth time of each passage (to a confluent monolayer) and the time to 75% CPE after viral inoculation were recorded. The harvested viral titers of each generation of passaged cells after viral inoculation were determined. The differences in ORFV replication in different generations of passaged cells were compared, and the growth conditions of the passaged cells were recorded.

## Abbreviations

CE: Contagious ecthyma
CPE: Cytopathic effects; FasL: Fas ligand
MDBK: Madin-Darby bovine kidney
ORFV: Orf virus
